# Reappraisal of telesurgery in the era of high‐speed, high‐bandwidth, secure communications: Evaluation of surgical performance in local and remote environments

**DOI:** 10.1002/ags3.12611

**Published:** 2022-08-12

**Authors:** Yoshiya Takahashi, Kenichi Hakamada, Hajime Morohashi, Harue Akasaka, Yuma Ebihara, Eiji Oki, Satoshi Hirano, Masaki Mori

**Affiliations:** ^1^ Department of Gastroenterological Surgery Hirosaki University Graduate School of Medicine Hirosaki Japan; ^2^ Committee for Promotion of Remote Surgery Implementation Japan Surgical Society Tokyo Japan; ^3^ Department of Gastroenterological Surgery II Hokkaido University Faculty of Medicine Sapporo Japan; ^4^ Department of Surgery and Science Kyushu University Fukuoka Japan; ^5^ Tokai University School of Medicine Isehara Japan

**Keywords:** communication delay, remote surgery, robotic surgery, telesurgery, video transmission delay

## Abstract

**Aim:**

Communication and video transmission delays negatively affect telerobotic surgery. Since latency varies by communication environment and robot, to realize remote surgery, both must perform well. This study aims to examine the feasibility of telerobotic surgery by validating the communication environment and local/remote robot operation, using secure commercial lines and newly developed robots.

**Methods:**

Hirosaki University and Mutsu General Hospital, 150 km apart, were connected via a Medicaroid surgical robot. Ten surgeons performed a simple task remotely using information encoding and decoding. The required bandwidth, delay time, task completion time, number of errors, and image quality were evaluated. Next, 11 surgeons performed a complex task using gallbladder and intestinal models in local/remote environments; round trip time (RTT), packet loss, time to completion, operator fatigue, operability, and image were observed locally and remotely.

**Results:**

Image quality was not so degraded as to affect remote robot operation. Median RTT was 4 msec (2‐12), and added delay was 29 msec. There was no significant difference in accuracy or number of errors for cholecystectomy, intestinal suturing, completion time, surgeon fatigue, or image evaluation.

**Conclusion:**

The fact that remote surgery succeeded equally to local surgery showed that this system has the necessary elemental technology for widespread social implementation.

## Graphical abstract

This study aims to examine the feasibility of telerobotic surgery by validating the communication environment and local/remote robot operation. The performance of remote surgery over a commercial line was equivalent to that of the local environment. Therefore, remote surgery using a commercial line can be performed safely, indicating that we have the elemental technology necessary for social implementation of telerobotic surgery.

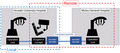

## 1 INTRODUCTION

Currently, surgical assist robots are deployed in a very large number of hospitals worldwide, and they are also becoming popular in Japan. There is a great demand for minimally invasive surgery, such as robotic surgery.^1^ Furthermore, there are high expectations for telerobotic surgery using commercial lines since optical fiber and 5G networks are available nationwide.^2^, ^3^ Telerobotic surgery has been studied since the 1970s, and the world's first tele‐laparoscopic cholecystectomy was performed between the US and France in 2001.^4^ This was followed by a number of remote surgeries in Canada,^5^ all of which were reported to be successful. However, many issues exist, such as the economics of dedicated communication lines, communication security, and communication delays, so widespread use has not progressed since those early experiments. In recent years, viable communication networks have been developed, and the issues of communication delay and security are being resolved. In addition, the development of new surgical robots has progressed, and various models are now available on the market.^6^ We conducted a demonstration of telerobotic surgery using a commercial line as a preliminary study in February 2021, and reported that it was possible to build a communication environment conducive to remote surgery.^7^ In August 2021, hinotori™️ (Medicaroid Corporation) was successfully operated remotely over a distance of 2000 km using a Science Information NETwork (SINET).^8^ However, since the communication delay and the video transmission information delay vary depending on the communication environment to be constructed and the robot to be used, it is necessary to verify communication environments and the robots involved in various combinations.

The purpose of this study is to examine the communication environment and robot operability in both local and remote circumstances using Japanese commercial lines and a new type of robot the, hinotori™️, already available on the market, as well as examine the potential for social implementation of telesurgery.

## 2 MATERIALS AND METHODS

### 2.1 Communication environment

Hirosaki University Hospital (Hirosaki City, Aomori, Japan) and Mutsu General Hospital (Mutsu City, Aomori, Japan), 150 km apart, were connected by a fiber optic network provided by Nippon Telegraph and Telephone East Corporation. The guaranteed bandwidth of the fiber optic network is 200 Mbps, and an IP‐VPN (Internet Protocol‐Virtual Private Network) was constructed. A guaranteed line is one in which the bandwidth can be selected according to the subscriber's purpose; and it comes with a service quality guarantee and 24‐hour/365‐day support service as standard. The data volume of the transmitted image information was limited to 120 Mbps by both the encoder and decoder. We evaluated the roundtrip time (RTT) of the network line, the delay times of the encoder and decoder, and the packet loss of the image during various telerobotic tasks.

### 2.2 Surgery robot

The robot used was the hinotori™️, a surgical assist robot developed by Medicaroid Corporation. The hinotori™️ consists of three units: the operation unit, the surgeon's cockpit, and the vision unit. The arms of the operation unit consist of eight joints that are designed to reduce interference between the arms. The surgeon's cockpit is operated by a hand controller, similar to the da Vinci surgical system, while viewing a closed 3D monitor.

**FIGURE 1:**
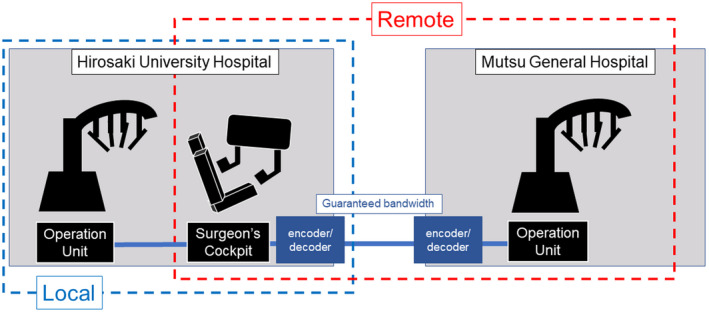
Hirosaki University Hospital and Mutsu general hospital, 150 km apart, were connected by a fiber optic network provided by Nippon Telegraph and Telephone east corporation. The data volume of the transmitted image information was limited to 120 Mbps by both the encoder and decoder.

In the remote state, the surgeon's cockpit and vision unit installed at Hirosaki University Hospital were connected to the operation unit installed at Mutsu General Hospital via the aforementioned line. In the local state, the task was performed at Hirosaki University Hospital connecting to an operation unit located in the same room (Figure 1).

### 2.3 Surgical tasks

Tasks were performed over a 5‐day period; peg transfer was performed during the first 1.5 days; simulated surgical tasks were performed throughout the remaining days; for simulated surgical tasks, one subject at a time performed a cholecystectomy and intestinal suturing in no particular order. All surgeons are gastroenterological surgeons, urologists, and gynecologists at Hirosaki University Hospital who have no vested interest in the robotics or telecommunications companies involved in the experiment and can evaluate the results fairly.

### 2.4 Peg transfer

The subjects were 10 surgeons with sufficient surgical experience, but mixed experience in robotic surgery, three had routinely used robotic surgery, and seven had no prior experience, who performed the relocation of a triangular prism remotely. This was a two‐handed task. Six Triangles™️^9^ were placed at designated locations on a peg board sitting on a table, which mechanically rocked to mimic respiratory fluctuation (TRP‐BHT1, by KOTOBUKI medical Inc.). For the background, we used a cloth printed with intra‐abdominal images of the upper abdomen, mainly the gallbladder, to simulate a real intraperitoneal environment. In follow‐up tasks, six triangular prisms were inserted into the rods on the left side, which were moved to the right side, or, conversely, six triangular prisms on the left side were moved to the right side. If the operator could not get hold of, dropped, or incorrectly inserted the triangles, that attempt was counted as an error.

### 2.5 Simulated surgical tasks

The subjects were 11 surgeons with extensive surgical experience (six of them were the same subjects as in the peg transfer. Three had routinely used robotic surgery, and eight had not.). Each of them performed two tasks, a cholecystectomy and intestinal suturing, under both non‐remote and remote conditions.

#### 2.5.1 Cholecystectomy

We used a gallbladder model manufactured by FASOTEC Co., Ltd. The right hand was used as a monopolar arm and the left hand as a bipolar arm that was dissecting using electrocautery. After identifying the cystic duct and the cystic artery, they were ligated proximally and distally, once each, with 2‐0 silk thread and separated. The gallbladder was then removed from the gallbladder bed to complete the task. The silk thread was provided and collected by the assistant. An evaluation method reported by Sharker et al was modified to evaluate the accuracy, number of errors, and time required.^10^ The number of errors was scored on a scale of 0‐6 for each ligation procedure. Accuracy was scored on a scale of 0‐6, with YES = 1 and NO = 0 for the six items.

#### 2.5.2 Intestinal suturing

**FIGURE 2:**
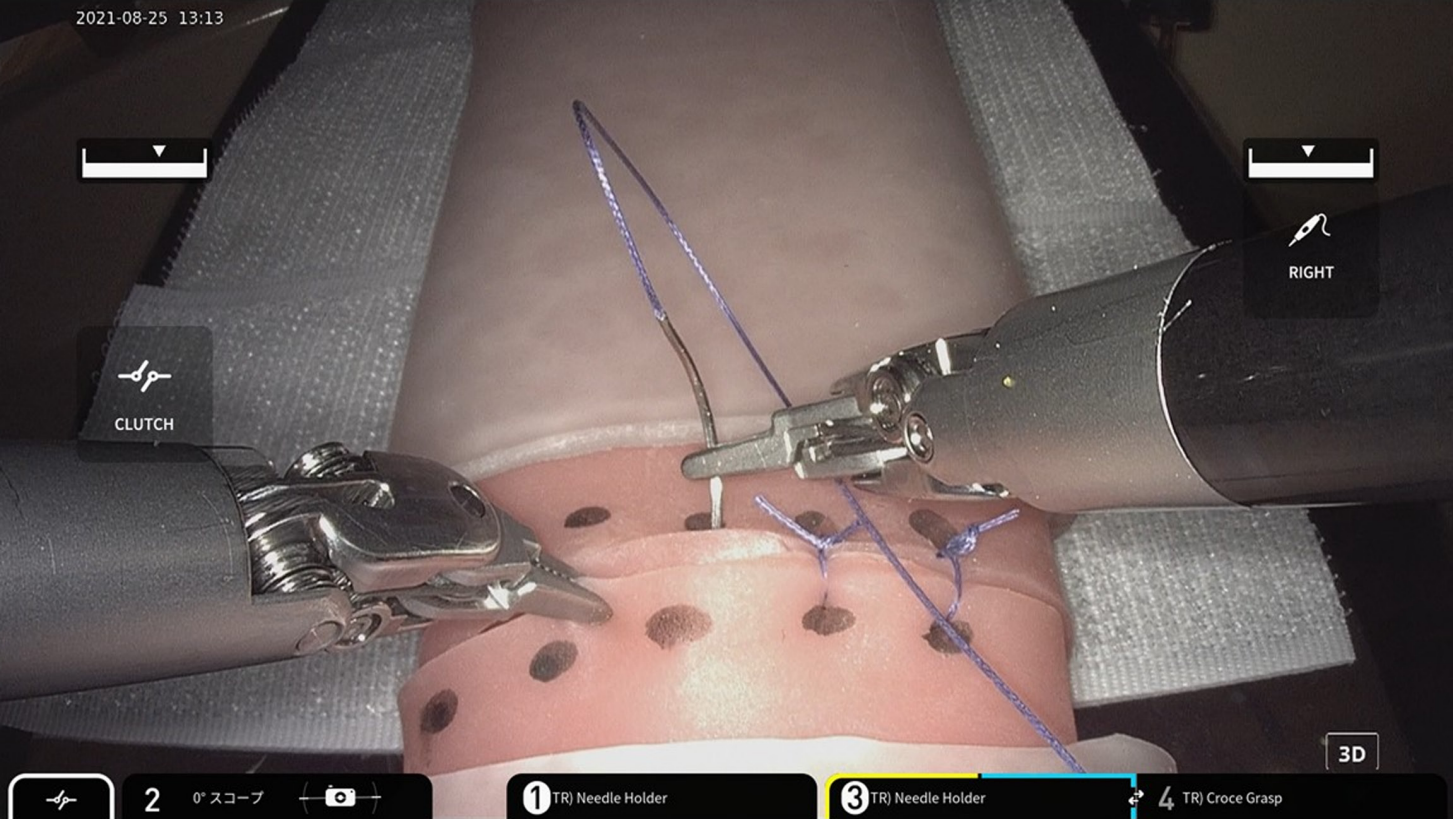
Five locations were marked at 5 mm intervals. Three single ligations were performed in one suture.

For intestinal suturing, we used an intestinal model made by AS ONE Corporation. Five suture sites were marked at 5 mm intervals, and three single ligations were performed in one suture (Figure 2). The time from the start of suturing to the fifth suture ligation and separation was measured and evaluated, and the accuracy and number of errors were evaluated by extracting the items that can be used for intestinal suturing using the evaluation method proposed by Goh et al.^11^ Accuracy was evaluated on a 5‐point scale (1‐5), and the score was 5‐25. The number of errors was evaluated using six items, and the cumulative value of the number of errors was used as the score.

### 2.6 Evaluation of image quality

Evaluation was performed after the completion of the remote and local tasks, respectively.

#### 2.6.1 Image quality score A

In order to analyze, in detail, the type of image quality degradation caused by changes in the communication environment and the impact of that on the procedure, we created a rating scale for clarity, stereoscopic vision, completeness, continuity, and impact on the procedure (Table S1). The image quality scale is a 5‐point scale, where a high score indicates that the image quality is not degraded and does not affect the procedure. The total score for each item was used for evaluation. A higher score means a higher rating and a lower score means a lower rating.

#### 2.6.2 Image quality score B

Image Quality Score B was created based on the image evaluation method proposed by Mitsuhashi.^12^ Specifically, an image quality rating scale was created to evaluate the degradation of image quality due to changes in the communication environment and its effect on the procedure (Table S2). The image quality rating scale is a 5‐point scale, where a high score indicates no degradation in image quality and no effect on the procedure. The median scores were compared. A higher score means a higher rating and a lower score means a lower rating.

### 2.7 Evaluation of robot operability

The mSUS and Robot Usability Score were assessed after the completion of the remote and local tasks, respectively. The PFS‐12 was administered both as a pre‐task and post‐task questionnaire.

#### 2.7.1 mSUS

The usefulness of remote surgery was assessed by mSUS, a modified version of The System usability scale (SUS) proposed by Brook.^13^ Nine items were each rated on a 5‐point scale, and the total score was calculated. A higher score means a higher rating and a lower score means a lower rating.

#### 2.7.2 Robot usability score

To evaluate the operability of the surgical robot, we modified the Robot Usability Score (Table S3).^14^ Eight items were rated on five levels, and the total score was calculated. A higher score means a higher rating and a lower score means a lower rating.

#### 2.7.3 PFS‐12

Subjective fatigue was assessed using the PFS‐12. Four items (behavioral, affective, sensory, and cognitive) were rated on a scale of 0‐10 and scored using the calculation method reported by Reeve et al.^15^ A higher score means greater fatigue and a lower score means less fatigue.

### 2.8 Statistical analysis

The software used was EZR.^16^ The normality test was the Kolmogorov‐Smirnov test, and when normality was not rejected, the paired *t* test was used. When normality was rejected, the Wilcoxon signed rank sum test was used. Statistical significance was determined at *P* < 0.05.

## 3 RESULTS

### 3.1 Transmission delay time

The RTT time was a median of 4 [2‐12] msec. The delay between encoder and decoder was 25 msec of the encoder‐decoder processing time. Therefore, the remote delay time compared to the local environment was 29 msec.

### 3.2 Video signal packet loss and surgical field images

When the line bandwidth during the surgical task was set to 200 Mbps, the bandwidth usage for transmission signals was 145 Mbps, and no packet loss was observed.

### 3.3 Evaluation of image quality

There was no significant difference in any of the five Image Quality Score A items, and no difference in total score (*P* = 0.07). There was a significant difference in Image Quality Score B, 4.91 (4, 5) in local and 4.45 (4, 5) in remote (*P* = 0.04) (Table 1).

**TABLE 1:** Comparison of image quality between local and remote

	Local	Remote	*P*
Image quality score A	23.9 (21‐25)	22.8 (20‐25)	0.07
Image quality score B	4.91 (4‐5)	4.45 (4‐5)	0.04*

*Note*: Values are averages (ranges).

* *P* < 0.05.

### 3.4 Evaluation of surgical performance

#### 3.4.1 Peg transfer

The study was validated at 200, 100, 80, and 70 Mbps line bandwidths. There were no significant differences in the task time or number of errors among the bandwidths. Similarly, no significant differences were found when the Image Quality Score was completed between the bandwidths (Table S4).

#### 3.4.2 Cholecystectomy task

The accuracy score was 5.0 (2.5‐6) for local and 4.9 (3.0‐6.0) for remote, and the number of errors was 1.0 (0‐2.5) for local and 1.1 (0‐3.5) for remote, with no significant difference (*P* = 0.82, *P* = 0.74) (Table 2). The completion time was 1156.3 (973‐1794) sec for local and 1247.3 (979‐1380) sec for remote, with no significant difference (*P* = 0.70). No differences in performance were observed among surgeons.

**TABLE 2:** Comparison of cholecystectomy task between local and remote

	Local	Remote	*P*
Accuracy (score)	5.0 (2.5‐6)	4.9 (3.0‐6.0)	0.82
Errors (score)	1.0 (0‐2.5)	1.1 (0‐3.5)	0.74
Total time (sec)	1156.3 (973‐1794)	1247.3 (979‐1380)	0.70

*Note*: Values are averages (ranges).

#### 3.4.3 Intestinal suturing task

The accuracy score was 22.8 (19.5‐25) for local and 22.5 (18.5‐25) for remote, and the number of errors was 1.4 (0‐4) for local and 1.8 (0‐5.5) for remote, with no significant difference (*P* = 0.43, *P* = 0.29). The completion time was 599.1 (350‐846) sec for local and 610.3 (327‐831) sec for remote, with no significant difference (*P* = 0.73) (Table 3). No differences in performance were observed among surgeons.

**TABLE 3:** Comparison of intestinal suturing task between local and remote

	Local	Remote	*P*
Accuracy (score)	22.8 (19.5‐25)	22.5 (18.5‐25)	0.43
Errors (count)	1.4 (0‐4)	1.8 (0‐5.5)	0.29
Total time (sec)	599.1 (350‐846)	610.3 (327‐831)	0.73

*Note*: Values are averages (ranges).

### 3.5 Evaluation of robot operability and surgeon fatigue

The total mSUS score was 35.5 (28‐44) for local and 34.6 (29‐44) for remote, and no significant difference was observed (*P* = 0.09) (Table 4). The total Robot Usability Score was 34.6 (26‐40) for local and 33.5 (27‐40) for remote (*P* = 0.30) (Table 5). No significant difference was found in any subscale. There was no significant difference in total score before and after the procedure as assessed by PFS‐12: 27.0‐30.9 (*P* = 0.15) for local and 31.5‐27.2 (*P* = 0.53) for remote. There was a significant difference in the “behavior” subscale from 2.2 to 2.9 (*P* = 0.01) in the local group (Table 6). No difference was found in the total score or in the degree of change for any subscale (Table S5).

**TABLE 4:** Evaluation of usefulness of remote surgery

	Local	Remote	*P*
mSUS	35.5 (28‐44)	34.6 (29‐44)	0.09

*Note*: Values are averages (ranges).

**TABLE 5:** Evaluation of robot operability

	Local	Remote	*P*
Physical comfort	4.1 (3‐5)	3.9 (3‐5)	0.42
Hand controls	4.4 (3‐5)	4.2 (3‐5)	0.42
Foot controls	4.0 (2‐5)	3.8 (2‐5)	0.59
3D vision	4.4 (4‐5)	3.9 (2‐5)	0.09
Annoyed or stressed	4.2 (2‐5)	4.1 (3‐5)	0.77
Smooth	4.6 (4‐5)	4.6 (4‐5)	1.00
Satisfaction	4.5 (4‐5)	4.5 (3‐5)	0.77
Actuality	4.5 (4‐5)	4.4 (4‐5)	0.35
Total	34.6 (26‐40)	33.5 (27‐40)	0.30

*Note*: Values are averages (ranges).

**TABLE 6:** Evaluation of surgeon fatigue

	Local			Remote		
	Before	After	*P*	Before	After	*P*
All score	27.0 (0.0‐64.0)	30.9 (2.0‐63.0)	0.15	31.5 (0.0‐82.0)	27.2 (0.0‐63.0)	0.53
Behavioral	2.2 (0.0‐6.0)	2.9 (0.7‐6.0)	0.01*	2.4 (0.0‐8.3)	2.7 (0.0‐7.3)	0.27
Affective	2.6 (0.0‐5.3)	2.9 (0.0‐6.7)	0.20	3.0 (0.0‐7.0)	2.5 (0.0‐6.0)	0.19
Sensorial	2.5 (0.0‐5.0)	2.4 (0.0‐5.0)	0.88	2.9 (0.0‐6.7)	2.2 (0.0‐5.0)	0.27
Cognitive	1.7 (0.0‐5.0)	2.0 (0.0‐5.0)	0.12	2.2 (0.0‐5.3)	1.7 (0.0‐5.0)	0.40

*Note*: Values are averages (ranges).

* *P* < 0.05.

## 4 DISCUSSION

As a result, the delay time added to the normal local environment was 29 msec; no packet loss was observed, and no degradation of image quality that would affect robot operation due to information encoding and decoding was apparent. We also demonstrated that robot operation in the local environment can be reproduced at the same performance level in a remote environment. The results of this study were elucidated using simulated surgical tasks similar to real clinical situations. Using an artificial organ model on a table with respiratory‐like movement, robotic surgery over commercial lines with next‐generation Japanese robots has the elemental technology necessary for the social implementation of remote surgery.

In general, telerobotic surgery suffers from problems related to communication and transmission delays that interfere with operations compared to what is expected with standard local surgery.^17^ There have been many reports on the extent to which communication delay is acceptable; Christopher et al^18^ reported on the validation of pyeloplasty in pigs using telerobotic surgery, and found that the average delay was 66.1 ± 1.5 msec, and no operability or visual impact on the surgeon was observed. It is generally believed that a delay time of 100 msec or more affects operability and makes surgery difficult.^19^, ^20^ In this study, the added delay in the remote environment was 29 msec, which was the sum of the communication delay due to the line and the delay due to the information processing in the encoder/decoder, compared to the normal local environment. It was an acceptable delay time that did not interfere with general surgery.

As for the evaluation of image quality, although no packet loss or fluctuation occurred in the communication environment, some subjects felt that the image quality was degraded. The cause was thought to be a reduction in image quality due to compression of the image information. We expect that the problem can be solved by improving information processing and transmission capabilities. This did not affect surgical procedures in the remote environment. Image quality information is mainly composed of three elements: resolution, color, and frame rate. In order to communicate image quality information, the information must be compressed by the encoder and decompressed by the decoder, and how and which of these elements are compressed and decompressed depends on the capabilities of the codec and the bitrate.^21^ The bitrate is the amount of data that can be sent and received in 1 second. The higher the bit rate, the more beautiful the image delivered, but the larger the amount of data to be transmitted, the more likely communication will be interrupted. In the future, the key to the success of remote surgery will be how to process and transmit these three elements of image information without causing stress to the surgeon.

There was no difference in surgical performance between local and remote tasks. In order to realize telerobotic surgery, it is necessary to verify not just simple tasks, but more complex and clinical tasks, which are similar to actual surgery, such as suturing and ligation, or removing organs using an electric scalpel. The fact that there is no difference in operability in complex tasks using the artificial organ model in this study is considered to be a major achievement toward widespread social implementation.

The mSUS score was mainly used to evaluate the usefulness of the robot system, while the Robot Usability Score was used to subjectively evaluate robot operability. However, none of the differences were significant between local and remote situations, including the subscales. There have been few reports describing detailed evaluation criteria for manipulability of the robot arm and image distortion.^6^ In this study, we made certain subjective and objective judgments using the various evaluation tables reported thus far and showed that there was no difference in the operability of the robot between remote and local tasks.

Previous studies using surgical robots have reported that surgical performance decreases when the workload is high.^22^ In the present study, a significant difference was found in the subscales regarding the degree of fatigue, but this was thought to be due to the overlapping number of times the task was performed, rather than fatigue due to remote operation. There was no difference in the other items or in the overall degree of change, and no significant feeling of fatigue was observed. However, it has been reported that more than half of surgeons experience physical fatigue after a long robotic surgery.^23^ In this study, the task was 20 minutes long at most, and it is necessary to examine how prolonged telerobotic surgery may affect the level of surgeon fatigue.

In Japan, a shortage of surgeons in rural areas exists due to the unequal distribution of doctors between urban and rural areas and the overall decrease in the number of surgeons. Telerobotic surgery is expected to provide an opportunity for young surgeons to work in rural areas, as it enables them to engage in local medical care in rural areas while providing surgical education and support for their supervisors at core hospitals.^1^, ^24^ In this environment, there are high expectations for telerobotic surgery, and early social implementation is required.

It would be ideal to have a communication line with a wide bandwidth that matches the required bandwidth of the robot. However, in general, communication lines using a wide bandwidth are extremely costly, and are likely to become a stumbling block to the social implementation and widespread use of telerobotic surgery. In the future, it is thought innovations such as narrowing down the amount of information in the robot signal, to match levels of communication bandwidth that are readily accessible, would be required as a way of economizing and thereby spreading the availability the telerobotic modality.

### 4.1 Limitations

Due to the limited verification time, the number of subjects was small, and it was not possible to randomly perform local and remote tasks. In addition, it was not possible to perform the validation blindly, although it was originally necessary to blindly perform validation of the subjects. However, it seems extremely important that we were able to evaluate the possibility of equivalent performance in a remote environment.

Only expensive leased lines with guaranteed bandwidth were used, and the verification did not take into account economical bandwidth alternatives or redundancy in case of communication interruptions. In addition, since only one type of communication bandwidth was set, there was no verification of behavior in situations where bandwidth was reduced.

## 5 CONCLUSIONS

The performance of remote surgery using hinotori™️ over a commercial line was equivalent to that of the local environment. Therefore, remote surgery with hinotori™️ using a commercial line can be performed safely, indicating that we have the elemental technology necessary for social implementation of telerobotic surgery.

## DISCLOSURE

Funding: This work was supported by a grant from the Japan Agency for Medical Research and Development (AMED), Grant Number JP21hs0122001h0002.

Conflict of Interest: Kenichi Hakamada, Eiji Oki, and Masaki Mori are an editorial board member of *Annals of Gastroenterological Surgery*.

Author Contributions: Y.T. did the study design, analyzed the data, interpreted the results, and wrote the manuscript. K.H. provided oversight and guidance for the entire study, interpreted and offered analyses of the results. H.M. and H.A. verified the study design, took charge of the data collection, interpreted, and offered analyses of the results. Y.E., E.O., S.H., and M.M. supervised this research. All authors reviewed the final manuscript.

## APPROVAL OF THE RESEARCH PROTOCOL AND INFORMED CONSENT

Because this research was not conducted on investigations that involve human subjects or laboratory animals, it was not necessary to obtain IRB approval.

## Supporting information


Appendix S1
