# Supplementary material for: Reappraisal of telesurgery in the era of high‐speed, high‐bandwidth, secure communications: Evaluation of surgical performance in local and remote environments
Source: Ann Gastroenterol Surg. 2022 Aug 12;7(1):167–74. doi: 10.1002/ags3.12611 (PMC9831893; doi:10.1002/ags3.12611)
Supplement: Supplementary file 1 — Appendix S1 [file AGS3-7-167-s001.docx]

Supplementary Table 1: Each of the five questions about image quality was evaluated on a five-point scale

**Image Quality Score A**

**Please answer the following questions about the surgical images of the robotic surgery you performed.**

On a scale of 1 to 5, with 5 being considered satisfactory for performing the procedure and 0 being considered completely unsuitable for performing the procedure, please rate the procedure.

**1. Clarity：How clear were the images from the robotic surgery?**

| It's not clear at all　　　　　　　　　　　　　　　　　　　　　　　　　　　Very clear | | | | |
| --- | --- | --- | --- | --- |
| 1 | 2 | 3 | 4 | 5 |

**2. Stereoscopic vision：Were the images of the robotic surgery in stereoscopic vision?**

| Was not stereoscopic vision at all　　　　　　　　　　　　　　Very stereoscopic vision | | | | |
| --- | --- | --- | --- | --- |
| 1 | 2 | 3 | 4 | 5 |

**3. Completeness：Was the robotic surgery screen complete?**

| Incomplete　　　　　　　　　　　　　　　　　　　　　　　　　　　　　　Complete | | | | |
| --- | --- | --- | --- | --- |
| 1 | 2 | 3 | 4 | 5 |

**4. Continuity：Were the robotic surgery screens continuous?**

| There's no continuity at all　　　　　　　　　　　　　　　　It's completely continuous | | | | |
| --- | --- | --- | --- | --- |
| 1 | 2 | 3 | 4 | 5 |

**5. Impact on the procedure：Were you able to perform the procedure with the images from this robotic surgery?**

| Could not perform at all　　　　　　　　　　　　　　　　　　Could be done perfectly | | | | |
| --- | --- | --- | --- | --- |
| 1 | 2 | 3 | 4 | 5 |

Total score:

Supplementary Table 2: The degradation of image quality was evaluated using a five-step evaluation scale

**Image Quality Score B**

**Please rate the quality of the surgical images of the robotic surgery performed this time on a scale of 1-5 according to the following criteria, and a continuous rating on a number line from 1-5.**

**5: I don't feel any degradation in image quality.**

**4: Image quality degradation is present, but not bothersome.**

**3: Image quality is degraded, but it does not interfere with surgery.**

**2: Image quality is degraded, but it does not interfere with surgery.**

**1: Image quality is degraded and I cannot perform surgery.**

**1)　Circle one of the numbers from 1-5.**

| 1 | 2 | 3 | 4 | 5 |
| --- | --- | --- | --- | --- |

**2)** **Mark on the number line from 1-5.**

**1　　　　　　　　　　　　　　　　　　　　5**

Supplementary Table 3: Eight items were rated on five levels

**Robot Usability Score**

Please answer the following questions about the tele-robotic surgery environment.

1: I don't think so at all 2: I don't think so 3: Neither　4: I think so 5: I strongly think so

| 1. I was physically comfortable. | | | | |
| --- | --- | --- | --- | --- |
| 1 | 2 | 3 | 4 | 5 |
|  |  |  |  |  |
| 2. I had good hand control in this environment. | | | | |
| 1 | 2 | 3 | 4 | 5 |
|  |  |  |  |  |
| 3. I had good foot control in this environment. | | | | |
| 1 | 2 | 3 | 4 | 5 |
|  |  |  |  |  |
| 4. I had a good 3D field of view in this environment. | | | | |
| 1 | 2 | 3 | 4 | 5 |
|  |  |  |  |  |
| 5. I had no complaints and felt little stress in this environment. | | | | |
| 1 | 2 | 3 | 4 | 5 |
|  |  |  |  |  |
| 6. The robot moved smoothly. | | | | |
| 1 | 2 | 3 | 4 | 5 |
|  |  |  |  |  |
| 7. The robot did exactly what I wanted it to do. | | | | |
| １ | 2 | 3 | 4 | 5 |
|  |  |  |  |  |
| 8. I think I can actually perform surgery using this type of robotic surgery environment. | | | | |
| 1 | 2 | 3 | 4 | 5 |
|  |  |  |  |  |
| 9. If you answered 1-3 to question 8, please choose the reason from the following. If you have any other reason, please provide it as a free answer.  ・The image was rough.　・The robot didn't work the way I wanted it to.  ・My technique was inexperienced.  ・Other（　　　　　　　　　　　　　　　　　　　　　　　　　　　　　） | | | | |

**Name：　　　　　　　　　Task No.　１・２　Total score：　 /40**

Supplementary Table 4: Comparison of Peg transfer. Values are averages (ranges).

|  | 200Mbps | 100Mbps | 80Mbps | 70Mbps |
| --- | --- | --- | --- | --- |
| Time (sec) | 74.2 (87.7-59.7) | 68.3 (87.7-49.3) | 61.9 (72.7-47.0) | 61.3 (74.0-44.3) |
| Errors (count) | 8.8 (19-3) | 7.8 (11-4) | 8 (14-4) | 8.6 (12-6) |
| Image Quality Score A | 22.3 (25-15) | 22.3 (25-16) | 22.2 (25-16) | 20.7 (24-14) |
| Image Quality Score B | 4.3 (5-3) | 4.1 (5-3) | 3.9 (5-3) | 3.4 (5-3) |

Supplementary Table 5: Degree of change in fatigue before and after the task. Values are averages (ranges).

| PFS-12 | ⊿Local | ⊿Remote | p |
| --- | --- | --- | --- |
| All score | 3.90 (-12-12) | -4.36 (-46-13) | 0.20 |
| Behavioral | 0.75 (-0.3-2.3) | 0.26 (-4.7-3) | 0.40 |
| Affective | 0.37 (-3-1.7) | -0.48 (-2.7-1) | 0.08 |
| Sensorial | -0.12 (-2-1.3) | -0.75 (-4.7-0.7) | 0.61 |
| Cognitive | 0.31 (-0.7-1) | -0.47 (-3.3-1) | 0.20 |
